# Preparing a global workforce for implementation of evidence‐based dementia care: facilitators and barriers

**DOI:** 10.1002/alz.092485

**Published:** 2025-01-09

**Authors:** Sokha Koeuth, Laura N. Gitlin, Justine S. Sefcik, Nancy A Hodgson, Michael Bruneau

**Affiliations:** ^1^ Drexel University, Philadelphia, PA USA; ^2^ Drexel University College of Nursing and Health Professions, Philadelphia, PA USA; ^3^ University of Pennsylvania, Philadelphia, PA USA

## Abstract

Dementia is a global public health challenge, impacting > 55 million individuals worldwide. However, widespread dissemination of efficacious non‐pharmacologic interventions remains limited. A key implementation barrier is workforce preparation and support for healthcare professionals (HPs) and administrators when being trained in, implementing, and sustaining programs. Normalization Process Theory (NPT) is a useful framework for understanding how complex interventions are perceived by frontline staff and integrated into daily work routines. NPT consists of four constructs: coherence (describing the process), cognitive participation (level of engagement of staff in the program), collective action (operational work), and reflective monitoring (perceived value of the program). Using NPT, we examined training and implementation barriers and facilitators for HPs and administrators from different countries embedding two evidence‐based programs (Tailored Activity Program (TAP) or Care of Older People in the Environment (COPE) in their settings.

Twenty‐nine participants (15 HP and 14 administrators) from the United States, Australia, Scotland, Italy, Chile, and Poland, were trained in either TAP or COPE, consisting of self‐paced online modules (10 hours for TAP; 16 hours for COPE), and accompanied by virtual meetings and personalized coaching. After training, participants used TAP/COPE with families in their respective countries and health systems. Seven focus groups (2‐4 participants per group) and eight individual interviews were conducted, using semi‐structured interview techniques, and qualitatively analyzed through directed content analysis to examine their experiences with these programs.

Applying NPT, we found general consensus that TAP/COPE were well received and perceived as beneficial to patients/clients (NPT = reflective monitoring). Nevertheless, additional resources for training and implementation were identified as important for effective implementation and sustainability: 1) strategies/checklists for getting started (NPT = coherence and cognitive participation), 2) support (webinars with seasoned implementers/trainers; NPT = cognitive participation and collective action), 3) and for the USA, billing information for reimbursement (NPT = collective action).

NPT provides a useful framework for understanding implementation and workforce preparatory challenges. We found that it is possible to effectively train and prepare a workforce using online, self‐paced approaches followed by live coaching opportunities. However, additional supports are needed for effective implementation/sustainability. A need for packaging evidence‐based programs in ways that facilitate implementation and sustainability is needed.

